# Identification of microRNAs Derived from Transposable Elements in the *Macaca mulatta* (Rhesus Monkey) Genome

**DOI:** 10.3390/genes14111984

**Published:** 2023-10-24

**Authors:** Eun Gyung Park, Yun Ju Lee, Jae-Won Huh, Sang-Je Park, Hiroo Imai, Woo Ryung Kim, Du Hyeong Lee, Jung-min Kim, Hae Jin Shin, Heui-Soo Kim

**Affiliations:** 1 Department of Integrated Biological Sciences, Pusan National University, Busan 46241, Republic of Korea; ehdtodt@pusan.ac.kr (E.G.P.); lsg5821@naver.com (Y.J.L.); dnfud647@pusan.ac.kr (W.R.K.); doo2080@naver.com (D.H.L.); jmk95@naver.com (J.-m.K.);; 2 Institute of Systems Biology, Pusan National University, Busan 46241, Republic of Korea; 3 National Primate Research Center, Korea Research Institute of Bioscience and Biotechnology, Cheongju 28116, Republic of Korea; huhjw@kribb.re.kr (J.-W.H.); parksj@kribb.re.kr (S.-J.P.); 4 Department of Functional Genomics, KRIBB School of Bioscience, Korea University of Science and Technology (UST), Daejeon 34113, Republic of Korea; 5 Molecular Biology Section, Center for the Evolutionary Origins of Human Behavior, Kyoto University, Inuyama, Aichi 484-8506, Japan; imai.hiroo.5m@kyoto-u.ac.jp; 6 Department of Biological Sciences, College of Natural Sciences, Pusan National University, Busan 46241, Republic of Korea

**Keywords:** *Macaca mulatta*, rhesus monkey, microRNA, transposable element, miRNAs-derived from TEs

## Abstract

Transposable elements (TEs) are mobile DNA entities that can move within the host genome. Over long periods of evolutionary time, TEs are typically silenced via the accumulation of mutations in the genome, ultimately resulting in their immobilization. However, they still play an important role in the host genome by acting as regulatory elements. They influence host transcription in various ways, one of which as the origin of the generation of microRNAs (miRNAs), which are so-called miRNAs derived from TEs (MDTEs). miRNAs are small non-coding RNAs that are involved in many biological processes by regulating gene expression at the post-transcriptional level. Here, we identified MDTEs in the *Macaca mulatta* (rhesus monkey) genome, which is phylogenetically close species to humans, based on the genome coordinates of miRNAs and TEs. The expression of 5 out of 17 MDTEs that were exclusively registered in *M. mulatta* from the miRBase database (v22) was examined via quantitative polymerase chain reaction (qPCR). Moreover, Gene Ontology analysis was performed to examine the functional implications of the putative target genes of the five MDTEs.

## 1. Introduction

microRNAs (miRNAs) are a class of small non-coding RNAs approximately 22 nucleotides in length [1,2]. Typically, miRNAs inhibit translation or induce mRNA degradation by binding to the 3′ untranslated region (3′ UTR) of the target messenger RNA (mRNA), acting as post-transcriptional regulators. miRNAs play a key role in the regulation of gene function and are involved in a variety of biological processes such as cell proliferation, differentiation, and angiogenesis [3,4]. In addition, dysregulation of miRNAs occurs during the progression of many diseases such as obesity and cancer [5,6]. Generally, miRNAs are transcribed from miRNA genes via RNA polymerase II. Nonetheless, some miRNAs originate from transposable elements (TEs), known as miRNAs derived from TEs (MDTEs) [7,8].

TEs are mobile DNA entities composed of repetitive sequences that induce genetic diversity via insertion into the host genome [9,10]. TEs are generally categorized into two classes based on their transposition intermediates [11]. Class I TEs are retrotransposons that insert into the host genome via reverse transcription of the RNA intermediate using a copy-and-paste mechanism [11,12]. Class I TEs are categorized into several types: long interspersed nuclear elements (LINEs), short interspersed nuclear elements (SINEs), and long terminal repeats (LTRs). Class II TEs are referred as DNA transposons that enter the host genome in the form of DNA segments using a cut-and-paste mechanism. Across extended periods of evolution, TEs tend to become quiescent due to the gradual accumulation of mutations within the genome, eventually leading to their immobilization. However, TEs continue to exert their effects on the host genome by functioning as regulatory elements. They influence host transcription in various ways, including serving as a source of miRNA generation. Some of the TEs have palindromic sequences that form miRNA hairpins, or the insertion of two similar TEs at adjacent positions within the genome can lead to the formation of the hairpin structures that could function as miRNAs [13,14,15]. It has been revealed that MDTEs are incorporated into the RNA-Induced Silencing Complex (RISC) by binding to Argonaute (AGO) proteins, and regulate gene expression in the same way as the other non-TE-derived miRNAs [16,17]. MDTEs can be divided into two categories based on the overlap between miRNAs and TEs. In the first case, TEs completely overlap with precursor miRNAs (pre-miRNAs). This results in the generation of two mature miRNAs from a single pre-miRNA, both of which originate from the same TE. In the second case, TEs partially overlap with pre-miRNAs, causing one of the two mature miRNAs to share a complete sequence overlap with the TE [18,19,20]. Although TEs are present in most prokaryotic and eukaryotic species, research on MDTEs remains limited [11].

In our previous study, we investigated MDTEs and their implication to human diseases. Herein, we extend our study on MDTEs to *M. mulatta*, one of the most extensively studied non-human primates (NHPs). *M. mulatta*, also known as the rhesus monkey or rhesus macaque, belongs to the subfamily Cercopithecinae of Old World monkeys and is native to South Asian countries [21,22]. They are an essential animal model for human health and disease research because of their high genetic proximity to humans (showing approximately 93% genome identity) and similarities in organ function to humans [23,24]. For these reasons, *M. mulatta* is considered a natural intermediate model that bridges the evolutionary and genetic gap between humans and the experimental rodents often used in human clinical trials [25,26,27]. The use of NHPs in experiments has led to important advances in biology and medicine because they play a crucial role in testing the safety of new drugs in human clinical trials [28]. In particular, *M. mulatta* made significant contributions during the COVID-19 pandemic as the most appropriate animal model for vaccine development [29]. However, despite its great contributions as a disease model organism, there are unbridgeable differences in genome sequences between *M. mulatta* and humans. Understanding and studying these differences could aid in drug development and safety testing. Genome-based analysis revealed that the differences in the genomes of these two species are found in non-coding regions, such as UTRs, and are not in the highly conserved protein-coding regions [24]. The sequence divergence of the UTRs (particularly within the 3′ UTR) causes alterations in gene expression patterns by modifying the binding sites for gene expression regulators, including transcription factors and miRNAs, ultimately contributing to the evolutionary dynamics [30]. Consequently, the sequence divergence also induces the expression alteration in these regulatory factors between two species [31]. Therefore, studying the differences in miRNAs between *M. mulatta* and humans can help researchers understand how they contribute to species-specific traits and adaptations. It may also provide an opportunity to overcome the challenges that interspecies differences can pose to drug testing for human clinical trials. However, a few studies have been conducted on miRNAs in *M. mulatta*, but no study on MDTEs.

In this study, we confirmed MDTEs within the genome of *M. mulatta* by intersecting the genomic coordinates of TEs and miRNAs. In addition, we compared identified *M. mulatta* MDTEs to human MDTEs based on our previous study [32]. As a result, we verified 68 common MDTEs between *M. mulatta* and humans and 17 MDTEs that were exclusively registered in *M. mulatta* based on the miRBase database (v22). We performed quantitative polymerase chain reaction (qPCR) to confirm the expression levels of 5 of 17 *M. mulatta*-specific MDTEs and conducted gene ontology (GO) and Kyoto Encyclopedia of Genes and Genomes (KEGG) pathway enrichment analysis to estimate their functional implications. Further studies are needed, but this research can contribute to the prediction and safety testing of novel therapeutics in preclinical trials by providing the first report of MDTEs that are exclusively present in the *M. mulatta* genome.

## 2. Materials and Methods

### 2.1. Identification of miRNAs Derived from Transposable Elements in M. mulatta Genome

To identify the genomic coordinates of *M. mulatta* miRNAs, the mml.gff3 file was downloaded from the miRBase v22 database (https://mirbase.org/ (accessed on 9 March 2023)) and a total of 990 mature miRNAs were registered. Among them, miRNAs labelled ‘JSUE’, of which the chromosomal location is uncertain, were excluded. Additionally, the multi-copy miRNAs produced from different chromosomes but having the same mature miRNA sequences were counted only once. Considering these two conditions, a total of 907 mature miRNAs remained. Using BedTools [33], the chromosomal locations of mature miRNAs were joined (intersectBed with options wa and wb) with the RepeatMasker output (BCM Mmul_8.0.1/rheMac8) downloaded from the UCSC table browser (https://genome.ucsc.edu/cgi-bin/hgTables (accessed on 9 March 2023)). Following the definition of MDTEs established in previous studies, we selected mature miRNAs that were completely covered by TE sequences as MDTEs [8,18]. Simple repeats and low-complexity elements were not considered in this study. According to the miRBase database (v22), five out of the 17 MDTEs identified only in *M. mulatta* were selected for further study, all with a read count of more than 100 from the deep sequencing results. The secondary structures and minimum free energy (MFE) of the pre-miRNAs were calculated using the RNAfold web server (http://rna.tbi.univie.ac.at/cgi-bin/RNAWebSuite/RNAfold.cgi (accessed on 12 March 2023)).

### 2.2. RNA Extraction and Complementary DNA (cDNA) Synthesis

Tissue samples from male and female *M. mulatta* were provided by the National Primate Research Center, Korea Research Institute of Bioscience and Biotechnology (KRIBB). Total RNA was isolated using Hybrid-R^TM^ (GeneAll, Seoul, Republic of Korea) according to the manufacturer’s instructions. Total RNA was quantified for both concentration and purity using an ND1000 UV-Vis spectrophotometer (NanoDrop Technologies, Wilmington, NC, USA). Then, the reverse transcription of total RNA was performed using an HB miR Multi Assay Kit^TM^ (SYSTEM I & SYSTEM II; HeimBiotek, Seoul, Republic of Korea) in accordance with the manufacturer’s recommendations. The conditions for cDNA synthesis were as follows: incubation at 37 °C for 60 min (SYSTEM I) or 50 °C for 60 min (SYSTEM II), followed by incubation at 95 °C for 5 min and holding at 4 °C.

### 2.3. Quantitative Polymerase Chain Reaction (qPCR)

To examine the relative expression of miRNAs, qPCR was performed using the HB_I Real-Time PCR Master Mix Kit (HeimBiotek, Seoul, Republic of Korea). Specific primers for miRNA amplification were designed and synthesized by HeimBiotek, Inc. Small nuclear RNA (snRNA) U6 was used to normalize the relative miRNA expression, and the same amount of each cDNA sample was used for the experiment. The PCR was performed on a Quantstudio1 system (Applied Biosystems, Foster City, CA, USA) and the conditions were as follows: hold at 95 °C for 15 min for initial activation, followed by 40 thermal cycles at 95 °C for 10 s and 60 °C for 40 s; standard melting conditions with 90 s at 55 °C and then 5 s each at 1 °C increments between 55 °C and 99 °C. The ramp rate of the last transformation of 60 °C to 95 °C was set at 0.15 °C/s. All samples were analyzed in triplicate and the relative expression data were analyzed using the 2^−ΔΔCt^ method.

### 2.4. Gene Ontology (GO) and Kyoto Encyclopedia of Genes and Genomes (KEGG) Pathway Enrichment Analysis

Target gene prediction for the miRNAs was performed using TargetScan Human 8.0 (https://www.targetscan.org/vert_80/ (accessed on 7 July 2023)), a web-based tool that computationally predicts miRNA targets. Predicted targets were required to have a total context ++ score of ≤−0.15 to reduce the number of false positives. Afterward, the selected target genes were subjected to GO and KEGG pathway analysis to examine the enrichment analysis using the web tool ShinyGO 0.80 (http://bioinformatics.sdstate.edu/go80/ (accessed on 3 October 2023)) with a false discovery rate (FDR) cut-off of 0.05. All tools were used with the species set to *M. mulatta*.

### 2.5. Statistical Analyses

Each experiment was conducted in triplicate, and the mean ± standard deviation (SD) of the data was plotted on a bar graph.

## 3. Results

### 3.1. Identification of MDTEs in the M. mulatta Genome

**Figure 1:**
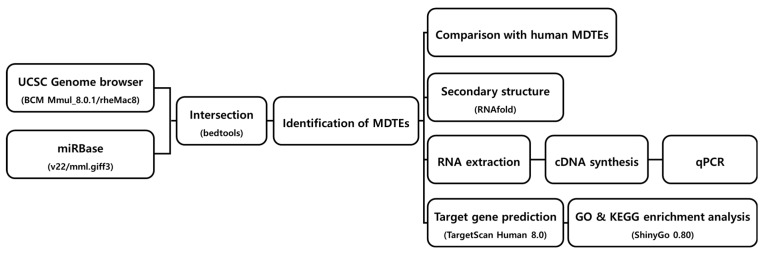
Workflow depicting the steps for MDTE identification and analysis.

**Figure 2:**
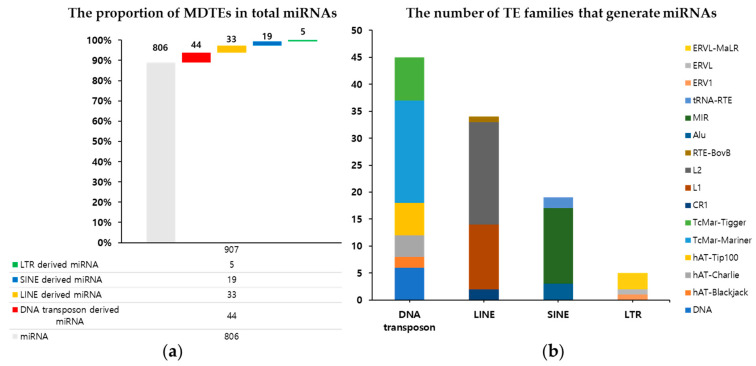
MDTE composition in *M. mulatta*. (**a**) The ratio of MDTEs among all miRNAs and the distribution of TE classes contributing to miRNA origin; (**b**) the proportion of TE families in each TE class that generates miRNAs.

The workflow of this study is illustrated in Figure 1. Briefly, we used the mml.gff3 file downloaded from the miRBase database (v22) to obtain the chromosomal coordinates of *M. mulatta* mature miRNAs and intersected them with the RepeatMasker output file containing the genomic coordinates of repeat sequences in the *M. mulatta* genome. Consequently, based on mature miRNAs, we confirmed the presence of 101 unique MDTEs in the *M. mulatta* genome. Detailed information on all the MDTEs is provided in Supplementary Table S1. Considering the total of 907 *M. mulatta* miRNAs registered in miRBase (v22), MDTEs accounted for approximately 11.1% of the total miRNAs (Figure 2a). Among the four TE classes, DNA transposons were most frequently responsible for MDTE generation, generating a total of 44 MDTEs. LINEs come second with 33 MDTEs, SINE with 19, and LTR with only five. At a more detailed level, the most abundant family of DNA transposons was TcMar-Mariner. For LINE, SINE, and LTR, it was the L2, MIR, and the ERVL-MaLR families, respectively. The detailed numbers of TE families constituting the MDTEs for each TE class are shown in Figure 2b and Supplementary Table S1.

### 3.2. Comparative Analysis of M. mulatta and Human MDTEs

In our previous investigation, we employed the same approach to identify MDTEs in the human genome and identified 352 miRNAs that were completely covered with TE sequences [34]. Here, we compared our findings on 101 MDTEs from *M. mulatta* and 352 MDTEs from humans to show how similar and different they are. There were 68 shared MDTEs between the two genomes (Table 1). Among the TE classes of miRNAs’ origin, the most overlapping class between the two genomes was DNA transposons, followed by LINEs, SINEs, and LTRs. Most miRNA origins were consistent with the level of TE names, but four miRNAs (miR-582-5p, miR-6130, miR-1304-5p, and miR-558) have different TE name origins. For instance, miR-582-5p originates from CR1-3_Croc in the CR1 family of the LINE class in rhesus, but in humans, it originates from L3 in the CR1 family.

Among the remaining 30 miRNAs, 10 were present in both *M. mulatta* and humans but were only derived from TEs in *M. mulatta*. Among these, miR-5697-5p and miR-5697-3p are registered in the miRBase database only for *M. mulatta* and humans. miR-4796-3p and miR-892b have been registered in chimpanzee and Bornean orangutan, which seems primate specific. The remaining four miRNAs (miR-151-5p, miR-151-3p, miR-421, and miR-507) are conserved across species. Apart from the three miRNAs (miR-548i-3p, miR-7177-5p, and miR-7177-3p) which are not registered in humans but are present in other species such as horses and common marmosets, we found 17 MDTEs that were only identified in *M. mulatta* according to the miRBase database (v22) (Table 2).

### 3.3. The Secondary Structures of Exclusively Existing MDTEs in M. mulatta

**Figure 3:**
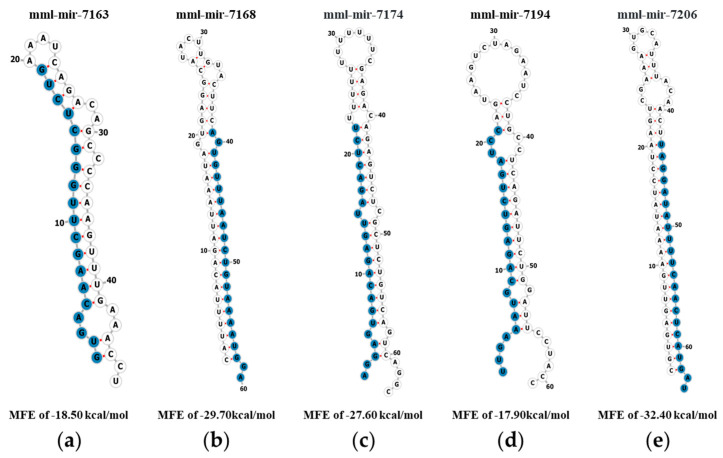
Prediction of the stem–loop structures and MFE values of MDTEs. The entire sequences of pre-miRNAs are represented and the mature sequences are indicated in blue. (**a**) mml-mir-7163; (**b**) mml-mir-7168; (**c**) mml-mir-7174; (**d**) mml-mir-7194; and (**e**) mml-mir-7206.

Since there is no experimental evidence or previous research on these 17 MDTEs, we examined their expression using quantitative polymerase chain reaction (qPCR). To narrow down the candidates for qPCR verification, criteria based on >100 read counts were used. As a result, five miRNAs (mml-miR-7163-5p, mml-miR-7168-3p, mml-miR-7194-5p, mml-miR-7174-5p, and mml-miR-7206-3p) were selected for further analysis. Before qPCR, the hairpin structures of the five MDTEs were predicted using pre-miRNA sequences obtained from the miRBase database (v22). All miRNAs exhibited a stem–loop configuration, as shown in Figure 3. In addition, minimum free energy (MFE) values were calculated to confirm the stability of the pre-miRNA structures. All putative pre-miRNA structures had MFE values lower than -17.90kcal/mol, with mml-mir-7206 being the most stable structure with the lowest MFE value of -32.40kcal/mol. This was followed by mml-mir-7168, mml-mir-7174, and mml-mir-7163, with mml-mir-7194 having the highest MFE value of −17.90 kcal/mol.

### 3.4. The Relative Expression Levels of MDTEs in M. mulatta Tissues

**Figure 4:**
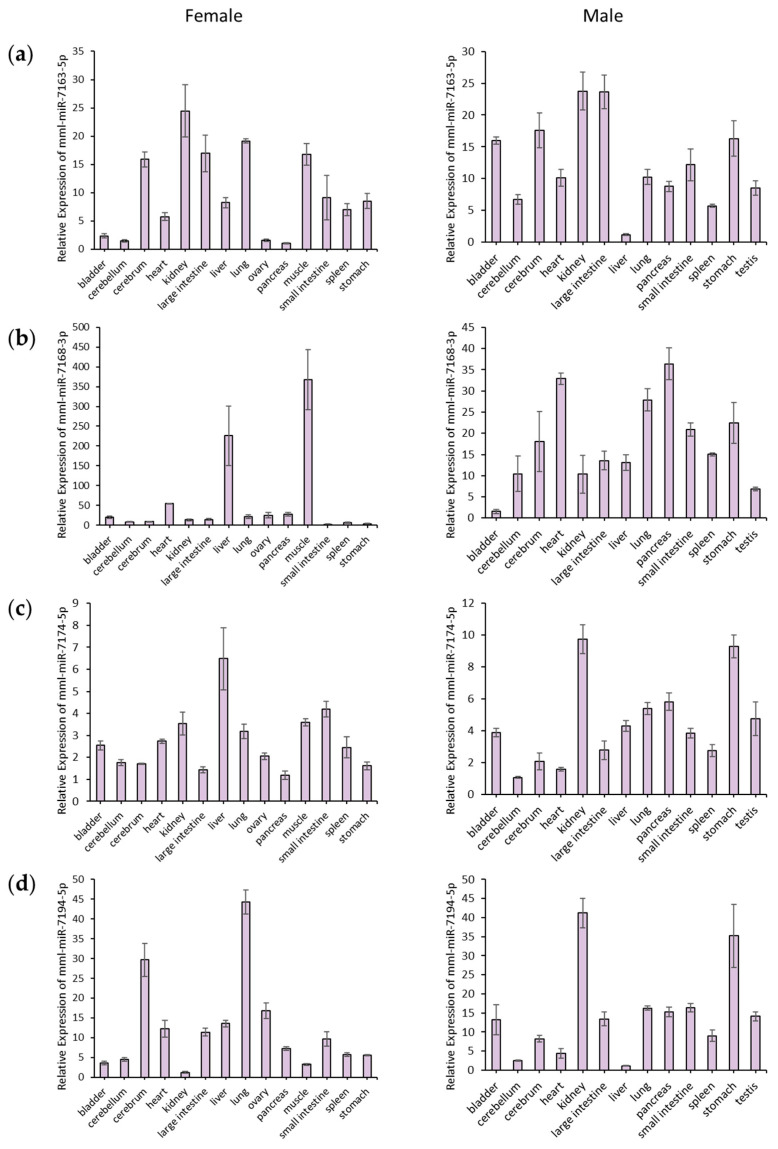

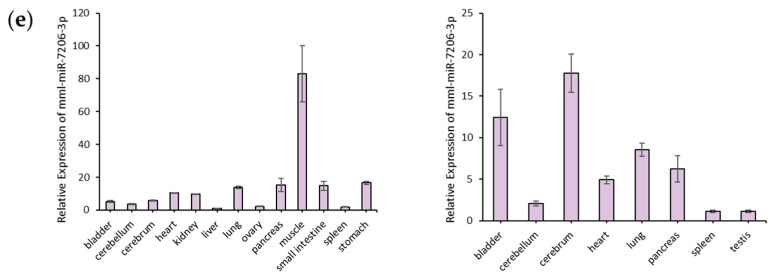
Relative expression levels of five MDTEs in a female and male *M. mulatta* tissues. (**a**) mml-miR-7163-5p; (**b**) mml-miR-7168-3p; (**c**) mml-miR-7174-5p; (**d**) mml-miR-7194-5p; and (**e**) mml-miR-7206-3p.

Given that no other study has validated the expression of these five MDTEs, we assessed their expression levels using qPCR in 14 tissues (e.g., bladder, cerebellum, cerebrum, heart, kidney, large intestine, liver, lung, ovary, pancreas, muscle, small intestine, spleen, and stomach) from a female *M. mulatta* and 13 tissues (e.g., bladder, cerebellum, cerebrum, heart, kidney, large intestine, liver, lung, pancreas, small intestine, spleen, stomach, and testis) from a male *M. mulatta* (Figure 4).

Among the MDTEs, mml-miR-7206-3p was not universally expressed across all tissues, and its expression pattern varied between a female and male. mml-miR-7206-3p was not detected in the large intestine of a female, whereas it was absent in the kidney, large intestine, liver, small intestine, and stomach of a male. Notably, the only tissue in which mml-miR-7206-3p was not expressed in either sex was the large intestine. Additionally, its expression was upregulated in female muscle and male cerebrum tissue.

In contrast, other miRNAs (e.g., mml-miR-7163-5p, mml-miR-7168-3p, mml-miR-7174-5p, and mml-miR-7194-5p) were expressed in all tissues of both sexes, with distinct expression patterns observed between a female and male. The expression of mml-miR-7163-5p was particularly prominent in the kidneys of both sexes. While showing high expression in all male tissues other than the liver, mml-miR-7163-5p displayed lower expression in the female bladder, cerebellum, ovary, and small intestine. The expression of mml-miR-7168-3p was specific to the liver and muscle of a female *M. mulatta*. In a male, it exhibited widespread expression except in the bladder and was particularly pronounced in the pancreas and heart. The expression of mml-miR-7174-5p was significantly higher in the liver of female *M. mulatta* and in the kidney and stomach of male *M. mulatta*. The expression of mml-miR-7194-5p was significantly high in the lung of female *M. mulatta*. Additionally, mml-miR-7194-5p was highly expressed in the kidney and stomach of a male, resembling the expression patterns of mml-miR-7174-5p in male.

### 3.5. GO and KEGG Pathway Enrichment Analysis of Target Genes Regulated by Five MDTEs

**Figure 5:**
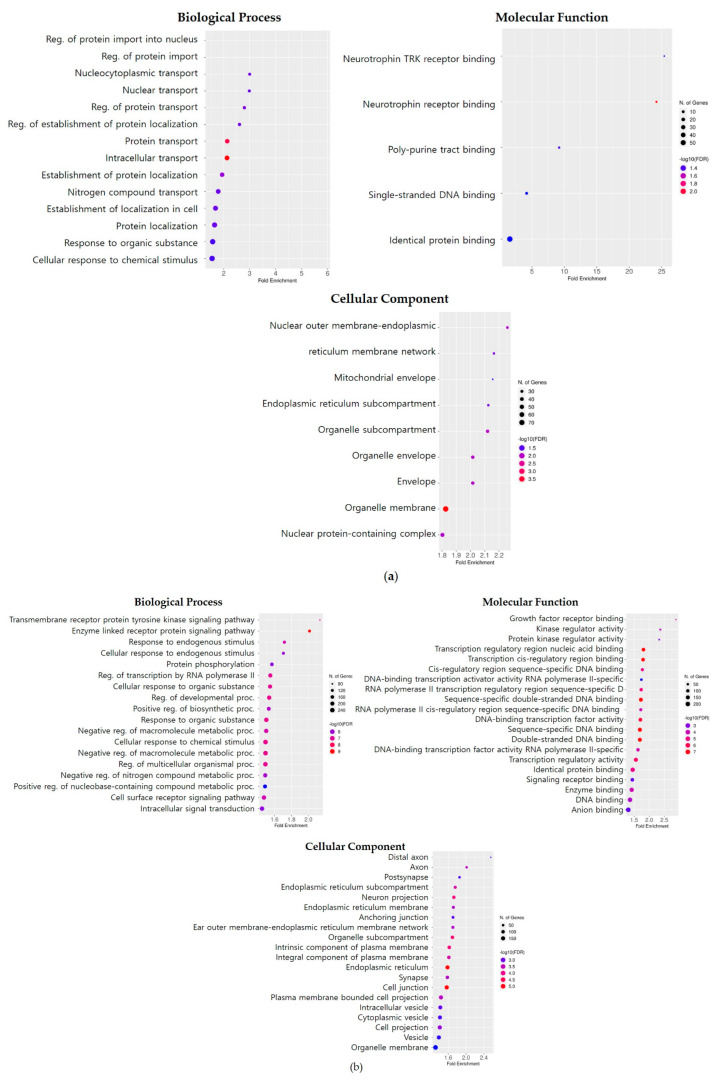

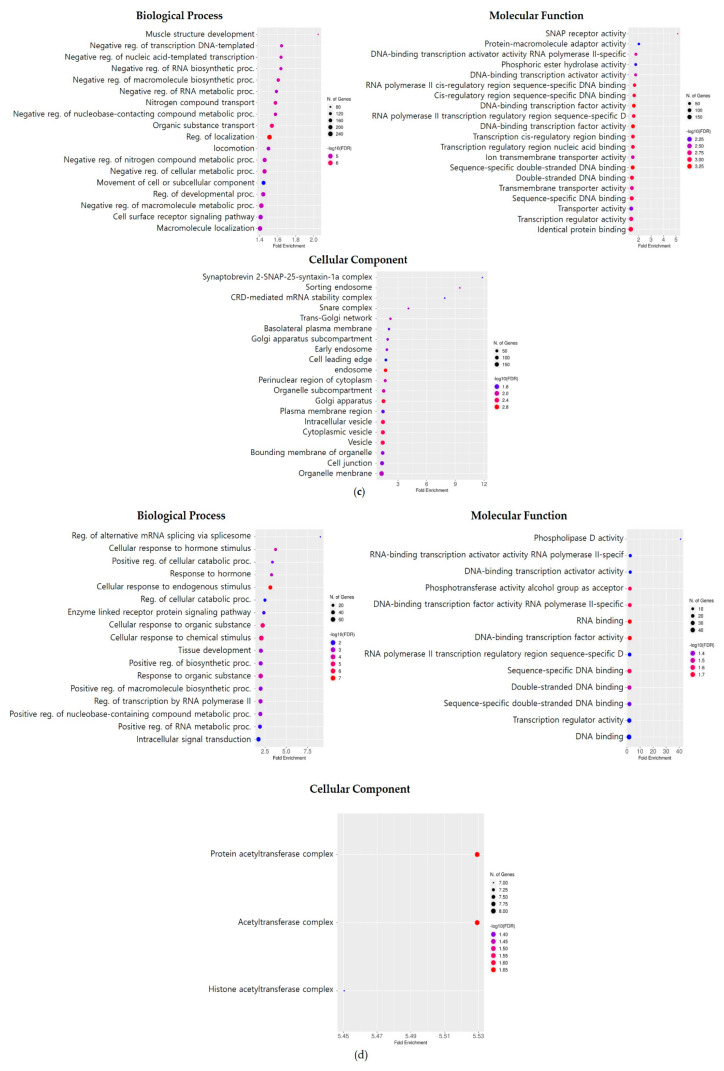

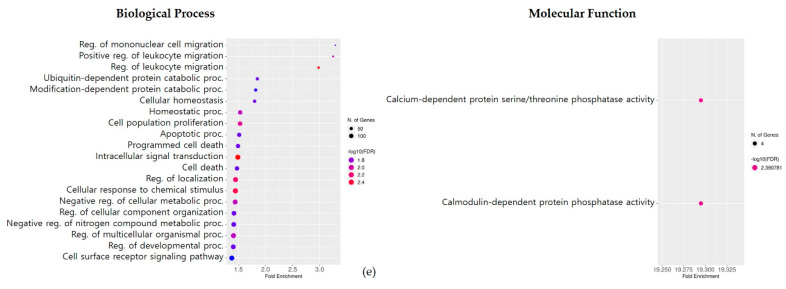
GO enrichment analysis about target genes of *M. mulatta* MDTEs. The known functions of genes are classified into three categories: Biological Process (BP), Molecular Function (MF), and Cellular Component (CC). The circle color represents −log10(FDR) and the circle size indicates the number of genes (FDR cut-off 0.05). (**a**) mml-miR-7163-5p; (**b**) mml-miR-7168-3p; (**c**) mml-miR-7174-5p; (**d**) mml-miR-7194-5p; and (**e**) mml-miR-7206-3p.

To examine the functional implications of these five MDTEs, their putative target genes were identified in *M. mulatta* using TargetScan Human 8.0. The predicted target genes for each miRNA were selected based on the criteria of a total context++ score ≤ −0.15 (Supplementary Tables S2–S6). Subsequently, GO enrichment analysis was conducted to understand the functional and statistical significance of the target genes influenced by the miRNAs of interest using the ShinyGO 0.80 database (FDR cut-off 0.05) (Figure 5). The known function of genes was organized into three main categories: biological processes (BP), molecular functions (MF), and cellular components (CC), and each group was evaluated separately.

The predicted target genes regulated by mml-miR-7163-5p were involved in various BP. Many genes were associated with transport and some exhibited significant enrichment in the regulation of protein import into the nucleus and the regulation of protein import in BP. In terms of MF, five binding pathways were identified and notably enriched in neurotrophin TRK receptor binding and neurotrophin receptor binding, exhibiting an enrichment of over 20 fold. Additionally, various genes associated with cellular organelles, particularly the endoplasmic reticulum, influenced the nuclear outer membrane–endoplasmic reticulum membrane network and the endoplasmic reticulum membrane in CC.

The target genes potentially affected by mml-miR-7168-3p were involved in numerous pathways in each category. Concerning the BP, these genes were associated with processes such as the transmembrane receptor protein tyrosine kinase signaling pathway and the enzyme-linked receptor protein signaling pathway. In MF, the genes were linked to functions like growth factor receptor binding, kinase regulatory activity, and protein kinase regulator activity. As for CC, the top three components with the highest fold enrichment were related to neuronal structures, including the distal axon, axon, and postsynapse.

The putative target genes of mml-miR-7174-5p were highly associated with muscle structure development in BP and SNAP receptor activity was overrepresented in MF. In terms of CC, four components were enriched more than 3 fold, including the synaptobrevin 2-SNAP-25-syntaxin-1a complex, sorting endosome, CRD-mediated mRNA stability complex, and SNARE complex.

The genes potentially influenced by mml-miR-7194-5p were significantly overrepresented in the regulation of alternative mRNA splicing via splicesome by more than 7.5 fold in BP and phospholipase D activity by more than 40 fold in MF. Regarding CC, the genes were associated with three components and were significantly enriched in the protein acetyltransferase complex and acetyltransferase complex.

The target genes likely regulated by mml-miR-7206-3p were associated with BP and MF, but not CC. The genes showed a tendency to be involved in migration-related processes, such as the regulation of mononuclear cell migration, the positive regulation of leukocyte migration, and the regulation of leukocyte migration in BP. Moreover, in MF, only two functions were overrepresented: calcium-dependent protein serine/threonine phosphatase activity and calmodulin-dependent protein phosphatase activity.

**Figure 6:**
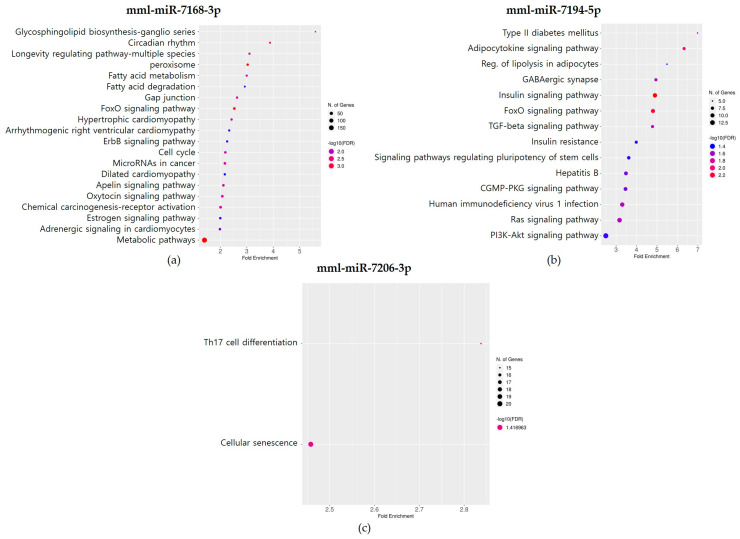
KEGG analysis of the putative target genes regulated by *M. mulatta* MDTEs. The circle color corresponds to −log10(FDR) and the circle size reflects the number of genes (FDR cut-off 0.05). (**a**) mml-miR-7168-3p; (**b**) mml-miR-7194-5p; and (**c**) mml-miR-7206-3p.

After conducting GO enrichment analysis, KEGG analysis was performed on the potential target genes of the five MDTEs with a total context++ score ≤ −0.15, similar to the GO enrichment analysis, to understand the high-level functions of the biological system (FDR cut-off 0.05). However, the putative target genes of two MDTEs, mml-miR-7163-5p and mml-miR-7174-5p, were not significantly involved in the biological pathways that meet the FDR cut-off of 0.05. As a result, no analysis results were obtained for these two MDTEs. The target genes of the remaining three MDTEs were subjected to KEGG pathway analysis and the results are presented in Figure 6. Analysis of the candidate target genes regulated by mml-miR-7168-3p revealed their involvement in 21 pathways. Notably, the top two pathways with the highest fold enrichment were the glycosphingolipid biosynthesis-ganglio series and circadian rhythm. In particular, the glycosphingolipid biosynthesis-ganglio series pathway exhibited a remarkable 5-fold enrichment. For mml-miR-7194-5p, its putative target genes were associated with 14 pathways. The most enriched pathways, type II diabetes mellitus and the adipocytokine signaling pathway, each showed over a 6-fold enrichment. KEGG analysis of the predicted target genes of mml-miR-7206-3p identified associations in only two pathways: Th17 cell differentiation and cellular senescence.

## 4. Discussion

*M. mulatta* is one of the most widely used non-human primate species in medical and biological research due to its similarities in physiology and organ function to humans [25,26,27]. To overcome the challenges that may arise in human clinical trials, it is important to understand the species-specific traits of the animal model, including the different gene expression responses regulated by differentially expressed miRNAs between *M. mulatta* and humans. Many miRNA gene families are evolutionarily conserved among mammalian species. It is considered that the same conserved miRNAs regulate similar pathways and biological processes among closely related species, such as primates [34,35]. However, MDTEs are more likely to represent species-specific traits than non-TE-derived miRNAs for some reasons: the insertion patterns of TEs in the genome is different across species, and some TE families are species-specific. In our previous study, we identified MDTEs in the human genome and investigated the diseases associated with them [32]. After classifying human MDTEs, we specifically focused on those derived from human endogenous retrovirus (HERV) sequences, which may represent human-specific properties. HERVs are a subfamily of LTR acquired from the human genome via exogenous retroviruses infection during primate evolution [36,37]. There were 29 HERV-derived miRNAs, and among them, 23 MDTEs were only found in humans [38]. Among the 23 HERV-derived miRNAs, hsa-miR-4454, which is derived from the HERV-H family, showed oncogenic traits by targeting the two tumor suppressor genes, *DNAJB4* and *SASH1*, in non-muscle-invasive bladder cancer (NMIBC).

In this study, we extended our research on MDTEs to *M. mulatta* and found 17 MDTEs that were only identified in *M. mulatta*. Among these 17 MDTEs, we conducted GO and KEGG pathway enrichment analysis of five MDTEs’ target genes to gain insights into their associated biological processes. From the KEGG pathway enrichment analysis, mml-miR-7168-3p and mml-miR-7194-5p were involved in various pathways related to diseases such as cancer, type II diabetes mellitus, and viral infection. mml-miR-7206-3p is associated with the innate immune system, the Th17 cell differentiation pathway. For the remaining two miRNAs, mml-miR-7163-5p and mml-miR-7174-5p, no significant enrichment was found from each of their target genes with the FDR cut-off of 0.05. However, with the FDR cut-off of 0.054, mml-miR-7163-5p showed a significant enrichment pathway related to nucleocytoplasmic transport. mml-miR-7174-5p, with the FDR cut-off of 0.051, showed three pathways including SNARE interactions in vesicular transport, adrenergic signaling in cardiomyocytes, and metabolic pathways (Supplementary Figure S1). The association of these species-specific miRNAs with disease and immune response-related pathways can be a significant challenge in drug testing and clinical trials.

Our study has some limitations as we identified MDTEs based on the genomic coordinates of miRNAs and TEs downloaded from the miRBase database and the UCSC table browser, respectively. With advances in sequencing technologies, many novel miRNAs have been identified in various species. However, our data are limited to miRNAs that are registered only in the miRBase database, and we cannot completely exclude the possibility that the 17 *M. mulatta*-specific MDTEs that we identified may also exist in other species. Nonetheless, despite these limitations, our study is important because it is the first to classify MDTEs in the *M. mulatta* genome and to provide experimental data for these MDTEs, the existence of which has been previously unproven. Further studies should be conducted to discern the specific functions of these MDTEs within *M. mulatta*, and investigating their regulatory roles would provide a better understanding of drug development for human clinical trials.

## Figures and Tables

**Table 1:** Common MDTEs between *M. mulatta* and humans.

Transposable Element		Rhesus			Humans		
Class	Family	Name	MDTEs	Sequence	Name	MDTEs	Sequence
DNA	DNA	MER135	miR-224-3p	AUGGUGCCCUAGUGACUACAA	MER135	miR-224-3p	AAAAUGGUGCCCUAGUGACUACA
			miR-224-5p	CAAGUCACUAGUGGUUCCGUUUA		miR-224-5p	UCAAGUCACUAGUGGUUCCGUUUAG
	hAT-Blackjack	MER81	miR-584-3p	AGUUCCAGGCCAACCAGGCUG	MER81	miR-584-3p	UCAGUUCCAGGCCAACCAGGCU
			miR-584-5p	UUAUGGUUUGCCUGGGACUGAG		miR-584-5p	UUAUGGUUUGCCUGGGACUGAG
	hAT-Charlie	MER5A1	miR-544	AUUCUGCAUUUUUAGCAAGUUC	MER5A1	miR-544a	AUUCUGCAUUUUUAGCAAGUUC
	hAT-Tip100	MER91C	miR-513a	UUCACAGGGAGGUGUCAUUUAU	MER91C	miR-513a-5p	UUCACAGGGAGGUGUCAU
			miR-513b	UUCACAAGGAGGUGUCAUUUAU		miR-513b-5p	UUCACAAGGAGGUGUCAUUUAU
			miR-652	AAUGGCGCCACUAGGGUUGUG		miR-652-3p	AAUGGCGCCACUAGGGUUGUG
	TcMar-Mariner	MARNA	miR-340-5p	UUAUAAAGCAAUGAGACUGAUU	MARNA	miR-340-5p	UUAUAAAGCAAUGAGACUGAUU
		MADE1	miR-548a	CAAUACUGGCAAUUACUUUUCC	MADE1	miR-548a-3p	CAAAACUGGCAAUUACUUUUGC
			miR-548b	CAAAAACCUCAAUUGCUUUUGU		miR-548b-3p	CAAGAACCUCAGUUGCUUUUGU
			miR-548c	CAAAACCGGCAAUUACUUCUGC		miR-548c-3p	CAAAAAUCUCAAUUACUUUUGC
			miR-548d-3p	CAAAAACCACAAUUUCUUUUGC		miR-548d-3p	CAAAAACCACAGUUUCUUUUGC
			miR-548d-5p	GGCAAAAACCACAAUUUCUUUU		miR-548d-5p	AAAAGUAAUUGUGGUUUUUGCC
			miR-548e	CAAAACCGGCAGUUACUUUUGC		miR-548e-5p	CAAAAGCAAUCGCGGUUUUUGC
			miR-548f	CAAAACCACAGUUCCUUUUGC		miR-548f-3p	AAAAACUGUAAUUACUUUU
			miR-548g-3p	GCAAAAACCAUAAUUACUUUUG		miR-548g-3p	AAAACUGUAAUUACUUUUGUAC
			miR-548g-5p	AGAGUAAUUGUGGUUUCUGUCA		miR-548g-5p	UGCAAAAGUAAUUGCAGUUUUUG
			miR-548h-3p	CAAAAACUGCAGUGACUUCUGU		miR-548h-3p	CAAAAACCGCAAUUACUUUUGCA
			miR-548h-5p	AGAAGUAAUUGCUGUUUUUGCC		miR-548h-5p	AAAAGUAAUCGCGGUUUUUGUC
			miR-548i-5p	AAAGGUCAUUGCAGAUUUUGCC		miR-548i	AAAAGUAAUUGCGGAUUUUGCC
			miR-548j-3p	AAAAACCGCAAUUACUUUUGC		miR-548j-3p	CAAAAACUGCAUUACUUUUGC
			miR-548j-5p	AAAAGUAAUUGUGGGCUUUGC		miR-548j-5p	AAAAGUAAUUGCGGUCUUUGGU
			miR-570	CAAAAGUAGCAAUUACCUUUGC		miR-570-3p	CGAAAACAGCAAUUACCUUUGC
			miR-579	UUCAUUUGGUACAAACCGCGAUU		miR-579-3p	UUCAUUUGGUAUAAACCGCGAUU
			miR-603	CACACACUGCAAUUACUUUUUC		miR-603	CACACACUGCAAUUACUUUUGC
	TcMar-Tigger	Tigger1	miR-1255a-5p	AGGAUGAGCAAUGGAAGUAGU	Tigger1	miR-1255a	AGGAUGAGCAAAGAAAGUAGAUU
			miR-1255b	UACGGAUAAGCAAAGAAAGUGG		miR-1255b-5p	CGGAUGAGCAAAGAAAGUGGUU
		Tigger18a	miR-4703-3p	GUAGUUGUACUGUAUUGCCACU	Tigger18a	miR-4703-3p	UGUAGUUGUAUUGUAUUGCCAC
		MER8	miR-649	AAACCUGUGUUGUUCAAGAGUC	MER8	miR-649	AAACCUGUGUUGUUCAAGAGUC
LINE	CR1	CR1-3_Croc	miR-582-5p	UUACAGUUGUUCAACCAGUUACU	L3	miR-582-5p	UUACAGUUGUUCAACCAGUUACU
		L3b	miR-582-3p	UAACUGGUUGAACAACUGAACC	L3b	miR-582-3p	UAACUGGUUGAACAACUGAACC
	L1	L1MA6	miR-6130	UGAGGGAGUGGAUUGUAUG	L1MA8	miR-6130	UGAGGGAGUGGAUUGUAUG
		L1MA9	miR-571	GAGUUGGCCACCUGGGUGAG	L1MA9	miR-571	UGAGUUGGCCAUCUGAGUGAG
		L1MB7	miR-562	AAAGUAGCUGUACCAUUUGC	L1MB7	miR-562	AAAGUAGCUGUACCAUUUGC
			miR-576-3p	AAGAUGUGGAAAAAUUGGAAUC		miR-576-3p	AAGAUGUGGAAAAAUUGGAAUC
			miR-576-5p	AUUCUAAUUUCUCCACAUCUUU		miR-576-5p	AUUCUAAUUUCUCCACGUCUUU
		L1MC4	miR-619	GAUCUGGACAUGUUUGUGCC	L1MC4	miR-619-3p	GACCUGGACAUGUUUGUGCCCAGU
		L1MCa	miR-625	GGACUGUAGAACUUUCUCCCU	L1MCa	miR-625-3p	GACUAUAGAACUUUCCCCCUCA
		L1MD2	miR-552	AACGGGUGACUGGUUAGACAA	L1MD2	miR-552-3p	AACAGGUGACUGGUUAGACAA
	L2	L2	miR-616	AAACCCUCCAAUGACU	L2a	miR-616-5p	ACUCAAAACCCUUCAGUGACUU
			miR-887	GUGAACGGGCGCCAUCCCGAGG		miR-887-3p	GUGAACGGGCGCCAUCCCGAGG
		L2a	miR-578	CUUCUUGUGCUCUGGGAUUGU		miR-578	CUUCUUGUGCUCUAGGAUUGU
		L2b	miR-1271-5p	CUUGGCACCUAGCAAGCACUCA	L2b	miR-1271-5p	CUUGGCACCUAGCAAGCACUCA
			miR-325	CCUAGUAGGUGUCCAGUAAGUGU		miR-325	CCUAGUAGGUGUCCAGUAAGUGU
			miR-493-3p	UGAAGGUCUACUGUGUGCCAGG		miR-493-3p	UGAAGGUCUACUGUGUGCCAGG
		L2c	miR-1249	ACGCCCUUCCCCCCCUUCUUCA	L2c	miR-1249-3p	ACGCCCUUCCCCCCCUUCUUCA
			miR-28-3p	CACUAGAUUGUGAGCUCCUGGA		miR-28-3p	CACUAGAUUGUGAGCUCCUGGA
			miR-374a-3p	CUUAUCAGAUUGUAUUGUAAUU		miR-374a-3p	CUUAUCAGAUUGUAUUGUAAUU
			miR-374b-3p	UUAGCAGGUUGUAUUAUCAUU		miR-374b-3p	CUUAGCAGGUUGUAUUAUCAUU
		L2d	miR-708-5p	AAGGAGCUUACAAUCUAGCUGGG		miR-708-5p	AAGGAGCUUACAAUCUAGCUGGG
		L2d2	miR-95-5p	UCAAUAAAUGUCUGUUGAAUU		miR-95-5p	UCAAUAAAUGUCUGUUGAAUU
	RTE-BovB	MamRTE1	miR-130a-3p	CAGUGCAAUGUUAAAAGGGC	MamRTE1	miR-130a-3p	CAGUGCAAUGUUAAAAGGGCAU
SINE	Alu	AluJr	miR-1304	UUCGAGGCUACAAUGAGAUGUG	AluJo	miR-1304-5p	UUUGAGGCUACAGUGAGAUGUG
	MIR	MIR	miR-607	GUUAUAGAUCUGGAUUGGAAC	MIR	miR-607	GUUCAAAUCCAGAUCUAUAAC
		MIR3	miR-6127	UGAGGGAGUGGGUGGGAGG	MIR3	miR-6127	UGAGGGAGUGGGUGGGAGG
		MIRb	miR-330-5p	UCUCUGGGCCUGUGUCUUAGGC	MIRb	miR-330-5p	UCUCUGGGCCUGUGUCUUAGGC
			miR-378d	ACUGGACUUGGAGUCAGAAGCA		miR-378d	ACUGGACUUGGAGUCAGAAA
			miR-633	CUAAUAGUAUCUACCACAAUAAA		miR-633	CUAAUAGUAUCUACCACAAUAAA
			miR-640	AUGAUCCAGGAACCUGCCUCU		miR-640	AUGAUCCAGGAACCUGCCUCU
		MIRc	miR-422a	ACUGGACUCAGGGUCAGAAGGC	MIRc	miR-422a	ACUGGACUUAGGGUCAGAAGGC
			miR-885-3p	AGGCAGCGGGGUGUAGUGGAUA		miR-885-3p	AGGCAGCGGGGUGUAGUGGAUA
	tRNA-RTE	MamSINE1	miR-342-3p	UCUCACACAGAAAUCGCACCCGU	MamSINE1	miR-342-3p	UCUCACACAGAAAUCGCACCCGU
			miR-342-5p	AGGGGUGCUAUCUGUGAUUGA		miR-342-5p	AGGGGUGCUAUCUGUGAUUGA
LTR	ERV1	MER101B	miR-924	AGAGUCUUGUGUUGUCUUGC	MER101B	miR-924	AGAGUCUUGUGAUGUCUUGC
	ERVL	LTR16D2	miR-3200-5p	GAAUCUGAGAAGGCGCACAAGGUUUGUG	LTR16D2	miR-3200-5p	AAUCUGAGAAGGCGCACAAGGU
	ERVL-MaLR	MLT1H2	miR-3927	CAGGUAGAUAUUUGAUAGGCA	MLT1H2	miR-3927-3p	CAGGUAGAUAUUUGAUAGGCAU
		MLT1C2	miR-558	UGAGCUGCUGUACCAAAAU	MLT1C	miR-558	UGAGCUGCUGUACCAAAAU

**Table 2:** The 17 MDTEs exclusively registered in *M. mulatta* based on miRBase.

miRNA					Transposable Element					
Chr	Start	End	miRNA	Read Counts	Chr	Start	End	Name	Class	Family
chr4	123669153	123669174	mml-miR-7187-3p	83	chr4	123669128	123669212	Tigger3c	DNA	TcMar-Tigger
chr5	100144152	100144173	mml-miR-1255a-3p	1	chr5	100142407	100144272	Tigger1	DNA	TcMar-Tigger
chr7	58404331	58404352	mml-miR-549b-5p	10	chr7	58404300	58404359	L2d2	LINE	L2
chr8	82810134	82810157	mml-miR-7198-5p	2	chr8	82809993	82810184	MLT1J1	LTR	ERVL-MaLR
chr11	3605770	3605788	mml-miR-7163-5p	251	chr11	3605601	3605797	MIRb	SINE	MIR
chr11	3605742	3605763	mml-miR-7163-3p	2	chr11	3605601	3605797	MIRb	SINE	MIR
chr17	22449738	22449759	mml-miR-7168-5p	2	chr17	22449731	22449783	MIR	SINE	MIR
chr17	22449700	22449721	mml-miR-7168-3p	311	chr17	22449685	22449729	MIR	SINE	MIR
chr17	23628698	23628718	mml-miR-7169-5p	0	chr17	23628444	23629215	L1ME1	LINE	L1
chr17	23628668	23628688	mml-miR-7169-3p	32	chr17	23628444	23629215	L1ME1	LINE	L1
chr17	29328120	29328140	mml-miR-7194-5p	153	chr17	29328075	29328230	MER5A1	DNA	hAT-Charlie
chr17	29328160	29328180	mml-miR-7194-3p	11	chr17	29328075	29328230	MER5A1	DNA	hAT-Charlie
chr18	2204518	2204539	mml-miR-7174-5p	696	chr18	2204270	2204539	AluJr	SINE	Alu
chr18	2204560	2204581	mml-miR-7174-3p	41	chr18	2204539	2204605	AluYRc0	SINE	Alu
chr18	24163604	24163624	mml-miR-7172-3p	10	chr18	24163467	24163633	MER5A	DNA	hAT-Charlie
chrX	19270373	19270394	mml-miR-7206-5p	0	chrX	19270357	19270416	Tigger5b	DNA	TcMar-Tigger
chrX	19270331	19270352	mml-miR-7206-3p	1604	chrX	19270286	19270368	Tigger5b	DNA	TcMar-Tigger

## Data Availability

The information about publicly available data were all described in Materials and Methods section.

## Supplementary Materials

The following supporting information can be downloaded at: https://www.mdpi.com/article/10.3390/genes14111984/s1, Figure S1: KEGG Analysis of predicted target genes regulated by *Macaca mulatta* MDTEs; Table S1: Genomic coordinates of miRNAs and overlapping TEs present in *M. mulatta*; Table S2: The predicted target genes of mml-miR-7163-5p; Table S3: The predicted target genes of mml-miR-7168-3p; Table S4: The predicted target genes of mml-miR-7174-5p; Table S5: The predicted target genes of mml-miR-7194-5p; Table S6: The predicted target genes of mml-miR-7206-3p.

## References

[1] Correia de Sousa M., Gjorgjieva M., Dolicka D., Sobolewski C., Foti M. (2019). Deciphering miRNAs’ action through miRNA editing. Int. J. Mol. Sci..

[2] Brites D. (2020). Regulatory function of microRNAs in microglia. Glia.

[3] Dasgupta I., Chatterjee A. (2021). Recent advances in miRNA delivery systems. Methods Protoc..

[4] Li X., Wang K., Ai H. (2019). Human umbilical cord mesenchymal stem cell-derived extracellular vesicles inhibit endometrial cancer cell proliferation and migration through delivery of exogenous miR-302a. Stem. Cells Int..

[5] Cirillo F., Catellani C., Sartori C., Lazzeroni P., Amarri S., Street M.E. (2019). Obesity, insulin resistance, and colorectal cancer: Could miRNA dysregulation play a role?. Int. J. Mol. Sci..

[6] Anvarnia A., Mohaddes-Gharamaleki F., Asadi M., Akbari M., Yousefi B., Shanehbandi D. (2019). Dysregulated microRNAs in colorectal carcinogenesis: New insight to cell survival and apoptosis regulation. J. Cell Physiol..

[7] Cui Y., Qi Y., Ding L., Ding S., Han Z., Wang Y., Du P. (2023). miRNA dosage control in development and human disease. Trends Cell Biol..

[8] Piriyapongsa J., Mariño-Ramirez L., Jordan I.K. (2007). Origin and evolution of human microRNAs from transposable elements. Genetics.

[9] Fueyo R., Judd J., Feschotte C., Wysocka J. (2022). Roles of transposable elements in the regulation of mammalian transcription. Nat. Rev. Mol. Cell Biol..

[10] Senft A.D., Macfarlan T.S. (2021). Transposable elements shape the evolution of mammalian development. Nat. Rev. Genet..

[11] Mat Razali N., Cheah B.H., Nadarajah K. (2019). Transposable elements adaptive role in genome plasticity, pathogenicity and evolution in fungal phytopathogens. Int. J. Mol. Sci..

[12] Grundy E.E., Diab N., Chiappinelli K.B. (2022). Transposable element regulation and expression in cancer. FEBS J..

[13] Smalheiser N.R., Torvik V.I. (2005). Mammalian microRNAs derived from genomic repeats. Trends Genet..

[14] Piriyapongsa J., Jordan I.K. (2007). A Family of Human MicroRNA Genes from Miniature Inverted-Repeat Transposable Elements. PLoS ONE.

[15] Yuan Z.D., Sun X.A., Jiang D.K., Ding Y., Lu Z.Y., Gong L.J., Liu H.D., Xie J.M. (2010). Origin and evolution of a placental-specific microRNA family in the human genome. BMC Evol. Biol..

[16] Ahn K., Gim J.A., Ha H.S., Han K., Kim H.S. (2013). The novel MER transposon-derived miRNAs in human genome. Gene.

[17] Ou-Yang F.Q., Luo Q.J., Zhang Y., Richardson C.R., Jiang Y.W., Rock C.D. (2013). Transposable element-associated microRNA hairpins produce 21-nt sRNAs integrated into typical microRNA pathways in rice. Funct. Integr. Genom..

[18] Yuan Z., Sun X., Liu H., Xie J. (2011). MicroRNA genes derived from repetitive elements and expanded by segmental duplication events in mammalian genomes. PLoS ONE.

[19] Qin S., Jin P., Zhou X., Chen L., Ma F. (2015). The role of transposable elements in the origin and evolution of microRNAs in human. PLoS ONE.

[20] Ghosh A., Platt R.N., Vandewege M.W., Tabassum R., Hsu C.-Y., Isberg S.R., Peterson D.G., Finger J.W., Kieran T.J., Glenn T.C. (2020). Identification and characterization of microRNAs (miRNAs) and their transposable element origins in the saltwater crocodile, Crocodylus porosus. Anal. Biochem..

[21] Wolfe L.D., Peters E.H. (1987). History of the freeranging rhesus monkeys (*Macaca mulatta*) of Silver Springs. Fla. Sci..

[22] Bunlungsup S., Kanthaswamy S., Oldt R.F., Smith D.G., Houghton P., Hamada Y., Malaivijitnond S. (2017). Genetic analysis of samples from wild populations opens new perspectives on hybridization between long-tailed (*Macaca fascicularis*) and rhesus macaques (*Macaca mulatta*). Am. J. Primatol..

[23] Gibbs R.A., Rogers J., Katze M.G., Bumgarner R., Weinstock G.M., Mardis E.R., Remington K.A., Strausberg R.L., Venter J.C., Wilson R.K. (2007). Evolutionary and biomedical insights from the rhesus macaque genome. Science.

[24] Ebeling M., Küng E., See A., Broger C., Steiner G., Berrera M., Heckel T., Iniguez L., Albert T., Schmucki R. (2011). Genome-based analysis of the nonhuman primate Macaca fascicularis as a model for drug safety assessment. Genome Res..

[25] Chiou K.L., Montague M.J., Goldman E.A., Watowich M.M., Sams S.N., Song J., Horvath J.E., Sterner K.N., Ruiz-Lambides A.V., Martínez M.I. (2020). Rhesus macaques as a tractable physiological model of human ageing. Philos. Trans. R. Soc. B.

[26] Yan G., Zhang G., Fang X., Zhang Y., Li C., Ling F., Cooper D.N., Li Q., Li Y., Van Gool A.J. (2011). Genome sequencing and comparison of two nonhuman primate animal models, the cynomolgus and Chinese rhesus macaques. Nat. Biotechnol..

[27] Koo B.-S., Lee D.-H., Kang P., Jeong K.-J., Lee S., Kim K., Lee Y., Huh J.-W., Kim Y.-H., Park S.-J. (2019). Reference values of hematological and biochemical parameters in young-adult cynomolgus monkey (*Macaca fascicularis*) and rhesus monkey (*Macaca mulatta*) anesthetized with ketamine hydrochloride. Lab. Anim. Res..

[28] Williamson B.N., Feldmann F., Schwarz B., Meade-White K., Porter D.P., Schulz J., Van Doremalen N., Leighton I., Yinda C.K., Pérez-Pérez L. (2020). Clinical benefit of remdesivir in rhesus macaques infected with SARS-CoV-2. Nature.

[29] Feng L., Wang Q., Shan C., Yang C., Feng Y., Wu J., Liu X., Zhou Y., Jiang R., Hu P. (2020). An adenovirus-vectored COVID-19 vaccine confers protection from SARS-COV-2 challenge in rhesus macaques. Nat. Commun..

[30] Suntsova M.V., Buzdin A.A. (2020). Differences between human and chimpanzee genomes and their implications in gene expression, protein functions and biochemical properties of the two species. BMC Genom..

[31] Dannemann M., Prüfer K., Lizano E., Nickel B., Burbano H.A., Kelso J. (2012). Transcription factors are targeted by differentially expressed miRNAs in primates. Genome Biol. Evol..

[32] Park E.G., Ha H., Lee D.H., Kim W.R., Lee Y.J., Bae W.H., Kim H.S. (2022). Genomic Analyses of Non-Coding RNAs Overlapping Transposable Elements and Its Implication to Human Diseases. Int. J. Mol. Sci..

[33] Quinlan A.R., Hall I.M. (2010). BEDTools: A flexible suite of utilities for comparing genomic features. Bioinformatics.

[34] Lee C.T., Risom T., Strauss W.M. (2007). Evolutionary conservation of microRNA regulatory circuits: An examination of microRNA gene complexity and conserved microRNA-target interactions through metazoan phylogeny. DNA Cell Biol..

[35] Friedman R.C., Farh K.K., Burge C.B., Bartel D.P. (2009). Most mammalian mRNAs are conserved targets of microRNAs. Genome Res..

[36] Seifarth W., Frank O., Zeilfelder U., Spiess B., Greenwood A.D., Hehlmann R., Leib-Mösch C. (2005). Comprehensive analysis of human endogenous retrovirus transcriptional activity in human tissues with a retrovirus-specific microarray. J. Virol..

[37] Grandi N., Tramontano E. (2018). HERV envelope proteins: Physiological role and pathogenic potential in cancer and autoimmunity. Front. Microbiol..

[38] Park E.G., Lee D.H., Kim W.R., Lee Y.J., Bae W.H., Kim J.M., Shin H.J., Ha H., Yi J.M., Cho S.G. (2023). Human Endogenous Retrovirus-H-Derived miR-4454 Inhibits the Expression of *DNAJB4* and *SASH1* in Non-Muscle-Invasive Bladder Cancer. Genes.

