# Supplementary material for: Identification of microRNAs Derived from Transposable Elements in the Macaca mulatta (Rhesus Monkey) Genome
Source: Genes (Basel). 2023 Oct 24;14(11):1984. doi: 10.3390/genes14111984 (PMC10671384; doi:10.3390/genes14111984)
Supplement: Supplementary file 1 [file genes-14-01984-s001.zip › supplementary figure1.pptx]

## Slide 1
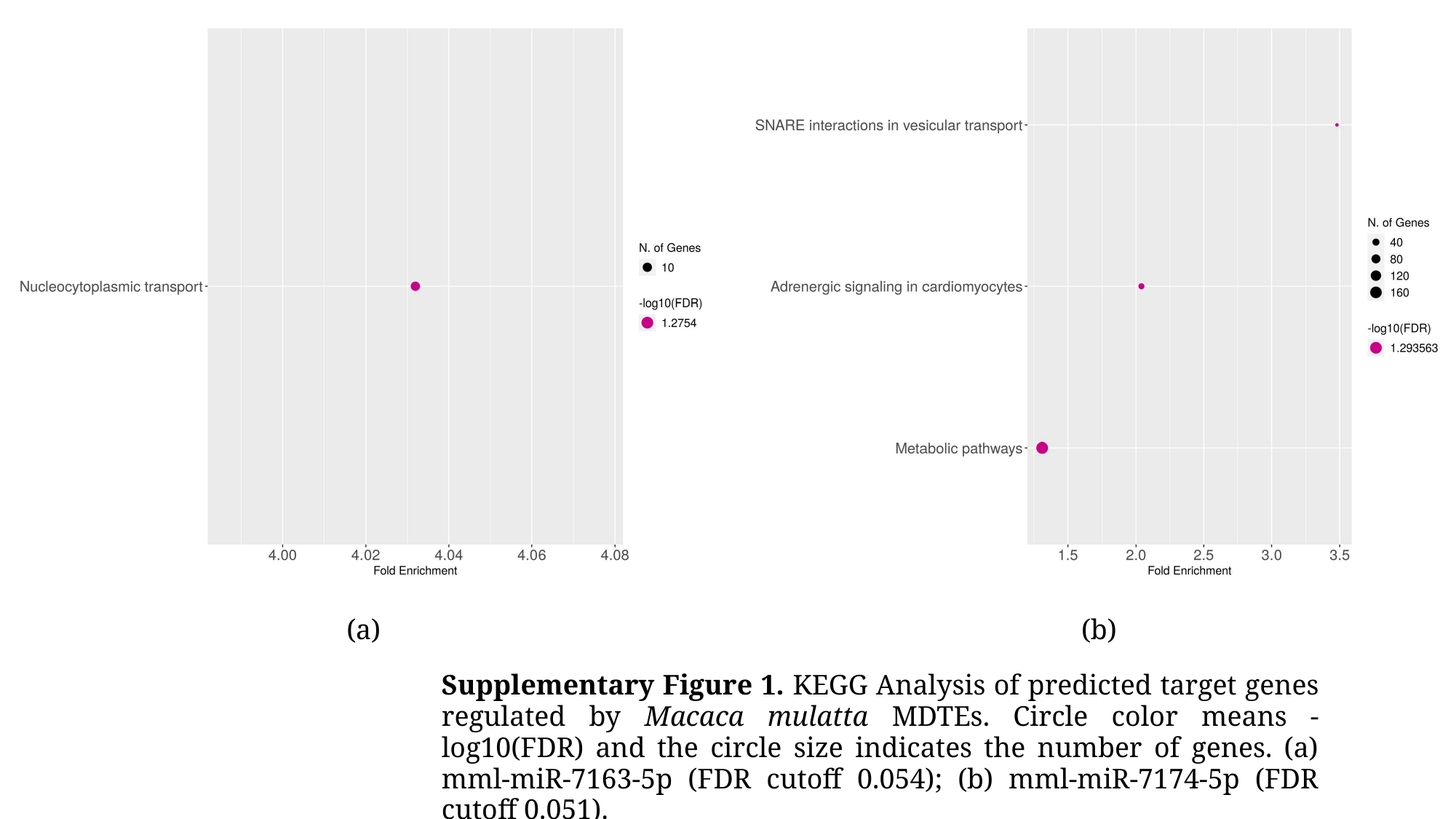

(a)
(b)
Supplementary Figure 1. KEGG Analysis of predicted target genes regulated by Macaca mulatta MDTEs. Circle color means -log10(FDR) and the circle size indicates the number of genes. (a) mml-miR-7163-5p (FDR cutoff 0.054); (b) mml-miR-7174-5p (FDR cutoff 0.051).
